# From Spatial Patterns to Prognosis: Decoding Single-Cell Architecture in Cancer with Hyperplex Immunofluorescence Imaging

**DOI:** 10.7150/jca.115037

**Published:** 2025-07-28

**Authors:** Mohammadreza Azimi

**Affiliations:** Institute of Microbiology and Virology, Riga Stradins University, LV-1067 Riga, Latvia.

**Keywords:** hyperplex IMF-based spatial proteomics, spatial biology, proximity analysis, cancer, precision oncology, cancer prognosis

## Abstract

Cancer prognosis relies not only on genetic and molecular biomarkers but also on the spatial organization of tumor and immune cells within the tumor microenvironment. Recent advances in spatial biology, particularly hyperplex immunofluorescence (IMF) imaging, have enabled high-dimensional, quantitative assessment of cell-cell interactions at the protein level. Nearest neighbor analysis (NNA) and proximity analysis have emerged as crucial computational methods for quantifying spatial distributions of tumor, stromal, and immune cells in hyperplex IMF datasets, providing insights into tumor heterogeneity, immune infiltration, and treatment response.

This review explores the current state of nearest neighbor and proximity analysis in cancer research, focusing on their applications in prognosis using single-cell spatial proteomics data generated by hyperplex IMF imaging. We summarize key computational approaches, including nearest neighbor distance metrics, Ripley's K-function, Voronoi tessellation, and graph-based models, that characterize spatial architecture within the tumor microenvironment. We highlight recent applications of hyperplex IMF in cancers showcasing how spatial proteomic signatures improve prognostic models. Furthermore, we discuss the integration of machine learning and AI-driven methods to leverage these spatial features for predictive modeling. Despite significant progress, challenges remain, including standardization of methodologies, variability in imaging technologies, and the need for large-scale, high-quality datasets. Addressing these challenges could lead to more accurate risk stratification and personalized treatment strategies.

By providing a comprehensive overview of nearest neighbor and proximity analysis in the context of hyperplex IMF-based spatial proteomics, this review aims to bridge the gap between computational methodologies and clinical applications, offering new perspectives on how spatial organization at the protein level influences cancer prognosis.

## Introduction

Cancer prognosis has traditionally relied on molecular and genetic biomarkers, such as driver mutations, gene expression profiles, and protein markers. However, recent advances in spatial biology have demonstrated that tumor progression is not only driven by intrinsic genetic alterations but also by the spatial organization of tumor cells, immune cells, and stromal components within the tumor microenvironment (TME). In particular, hyperplex immunofluorescence (IMF) imaging now enables highly multiplexed, spatially resolved proteomic profiling, allowing for comprehensive single-cell protein analysis within intact tissues.

Nearest neighbor analysis (NNA) and proximity analysis have emerged as powerful computational techniques for quantifying spatial relationships between cells, enabling researchers to study tumor-immune interactions 1, clonal evolution 2, and microenvironmental influences on therapy response 3 (Table 1). These spatial analyses provide a quantitative framework for understanding how cellular positioning influences prognosis, offering new avenues for precision oncology 4.

The concept that spatial patterns within the TME influence prognosis has been supported by several studies 5-17. For example, the spatial proximity of CD8⁺ T cells to tumor cells has been associated with improved survival in multiple cancer types, including colorectal 18 and lung cancer 19. Tumors with high infiltration of cytotoxic T cells (tumor-infiltrating lymphocytes, TILs) in close proximity to cancer cells often have a better prognosis. Conversely, exclusion of cytotoxic T cells from the tumor core is typically linked to poor prognosis and resistance to immune checkpoint blockade therapy 20. Tumors with spatially segregated immune populations may exhibit immune evasion mechanisms that contribute to worse clinical outcomes. Similarly, the presence and organization of macrophages 21, fibroblasts 22, and endothelial cells 23 can determine whether the TME supports tumor growth or acts as a barrier to progression.

In addition to immune infiltration, tumor heterogeneity—a hallmark of cancer—can also be captured using spatial proteomics. Tumors with highly heterogeneous spatial cell distributions tend to be more aggressive and resistant to treatment. Cancer is inherently a spatially heterogeneous disease, characterized by regions of varying cellular density, differential immune cell infiltration, and diverse tumor subclones that evolve under selective pressures. The spatial distribution of cancer cells and their microenvironmental components can directly influence tumor aggressiveness, metastatic potential, and therapy response 24.

Several landmark studies have recently leveraged single-cell and spatial transcriptomics to map tumor-immune architectures across cancer types 25-29. These studies have revealed how spatial organization of tumor-associated fibroblasts, immune-suppressive niches, and clonal expansions impact prognosis and treatment response. While these findings underscore the power of spatially resolved single-cell data, the potential of spatial proteomics, particularly using hyperplex IMF, to capture protein-level interactions in situ has been less extensively reviewed. Our review therefore complements and extends this body of work by focusing on spatial proteomic profiling of the tumor microenvironment in cancer prognosis.

**Figure 1:**
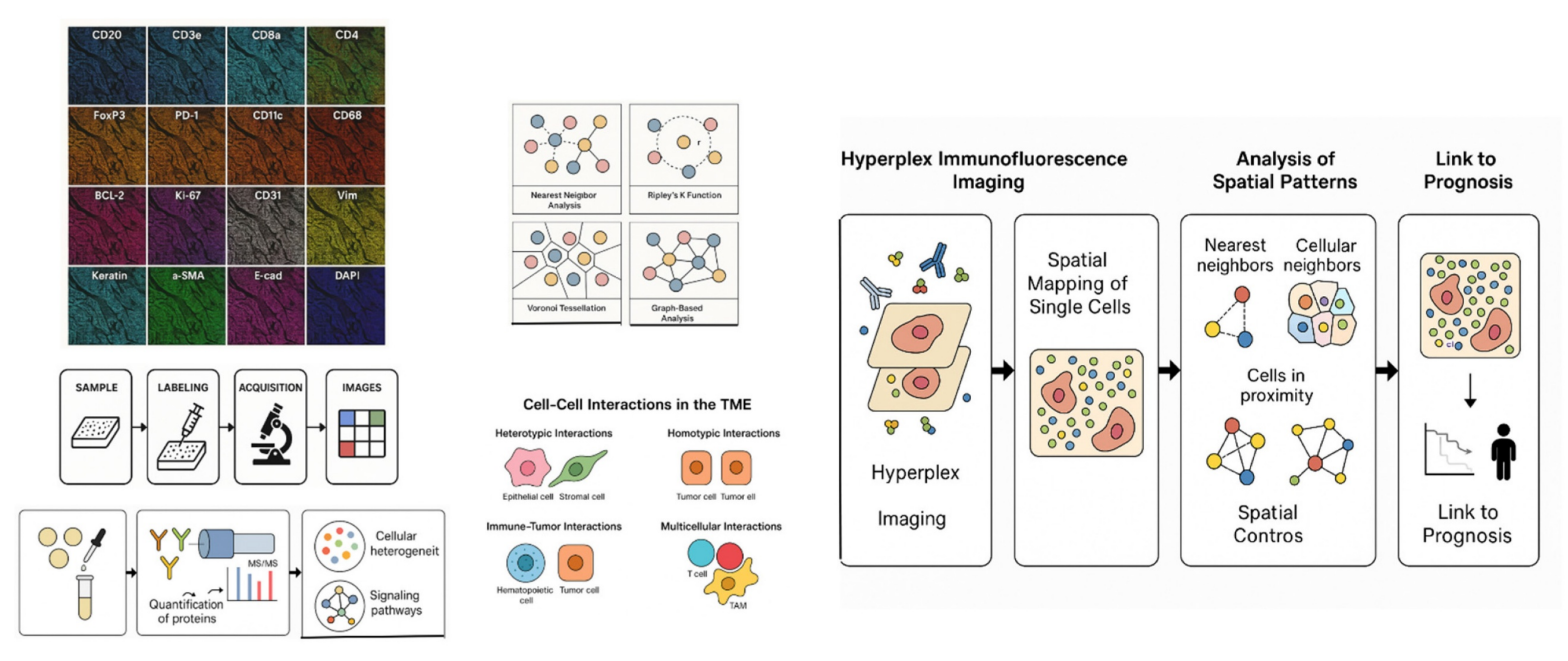
By providing quantitative, high-dimensional mapping of protein expression across thousands of individual cells within the natural tissue architecture, hyperplex IMF-based spatial proteomics might help unravel complicated spatial patterns. In order to decipher the spatial proteomic architecture of tumors and its influence on prognosis, the pipeline investigates the use of closest neighbor and proximity analysis frameworks to hyperplex IMF data.

Hyperplex IMF-based spatial proteomics offers a powerful approach to deciphering these complex spatial patterns by enabling quantitative, high-dimensional mapping of protein expression across thousands of individual cells within their native tissue architecture. This review explores how nearest neighbor and proximity analysis frameworks can be applied to hyperplex IMF data to decode the spatial proteomic architecture of tumors and its impact on prognosis. We highlight recent studies leveraging hyperplex IMF to reveal spatial heterogeneity in cancers such as breast, lung, and colorectal cancer, and discuss emerging computational tools and challenges in integrating spatial features into predictive models (Figure 1). By synthesizing current methodologies and clinical implications, we aim to provide a comprehensive overview of how single-cell spatial proteomics can inform cancer risk stratification and precision oncology.

## Multiplexed Imaging and Neighborhood Analysis

Artificial intelligence (AI) and machine learning (ML) approaches have been integrated into spatial analysis workflows. AI models can detect subtle spatial patterns that may not be apparent to human observers, improving prognostic accuracy. Deep learning techniques applied to histopathology slides, coupled with nearest neighbor and proximity-based spatial features, have demonstrated potential in predicting response to immunotherapy and chemotherapy 30.

Nearest neighbor analysis (NNA) 31 is a statistical method used to assess the spatial relationships between objects in a given space. In cancer research, NNA is commonly applied to quantify the distance between tumor cells, immune cells, and stromal components, allowing for a detailed characterization of the tumor microenvironment. Proximity analysis 32 expands on this concept by incorporating a broader range of spatial features, including cell clustering, cellular niches, and local density variations. Nearest neighbor and proximity analysis provide powerful tools for studying the spatial organization of tumors and their microenvironments. By quantifying how tumor and immune cells are arranged within tissue, these methods offer valuable prognostic insights and have the potential to inform precision medicine strategies. The integration of spatial proteomics, high-resolution imaging, and AI-driven computational approaches will likely enhance our ability to predict cancer progression and response to treatment.

By applying spatial statistics such as Voronoi tessellation 33 and Ripley's K-function 34, it is possible to assess clonal expansion, regional differences in biomarker expression, and microenvironmental influences on tumor evolution. However, addressing challenges related to data standardization, biological interpretation, and clinical validation will be critical for translating these methods into routine clinical practice.

In recent years, advances in multiplexed imaging and single-cell analysis have facilitated the study of spatial relationships within tumors at unprecedented resolution. Technologies such as multiplex immunohistochemistry (mIHC) 35, multiplex immunofluorescence (mIF) 36, Imaging Mass Cytometry (IMC) 37, and spatial transcriptomics 38 allow researchers to map cellular interactions in situ, revealing how spatial positioning influences tumor biology. Multiplexed imaging techniques such as CODEX (CO-Detection by Indexing) 39 and CELLDive 40 allow researchers to simultaneously measure dozens of proteins in tissue sections while preserving spatial information 41. These methods provide an unprecedented opportunity to study how different cell types interact within the TME and how these interactions contribute to patient outcomes and the integration of computational spatial analysis methods, such as nearest neighbor distance calculations, graph-based approaches, and clustering algorithms, has further enhanced our ability to interpret spatial data in the context of prognosis 41.

It is important to recognize that the spatial patterns identified through nearest neighbor and proximity analysis can have distinct features and consequences in differentiated versus stem-like cell populations. In differentiated cells, nearest neighbor interactions often reflect the structural and functional organization of the tumor microenvironment, such as the spatial segregation of immune cell subsets or the clustering of tumor cells with supportive stroma. In contrast, in stem-like or progenitor cell populations, spatial interactions may indicate niches that support self-renewal, therapy resistance, and metastatic potential. For instance, cancer stem cells that are closely associated with specific stromal or immune cell types may exhibit unique survival advantages, driving disease progression and recurrence. Accounting for these differences is crucial for interpreting the prognostic relevance of spatial patterns and for designing targeted therapeutic strategies 42.

Despite significant progress, challenges remain in applying nearest neighbor and proximity analysis in clinical oncology. One of the major obstacles is the lack of standardization in computational pipelines. Different studies use varying definitions of spatial metrics, making it difficult to compare results across datasets. Furthermore, spatial analysis is highly dependent on data quality, including the resolution of imaging techniques and the accuracy of cell segmentation algorithms. Another challenge is the interpretation of spatial data in a biological context. While computational methods can identify statistically significant spatial patterns, their functional relevance is not always clear. For example, does clustering of a particular immune cell type indicate an active anti-tumor response, or does it reflect immune exhaustion? 20.

Addressing these questions will be essential for translating spatial proteomic insights into meaningful clinical applications.

## Prognosis and Spatial Analysis

The spatial distribution of immune cells and their proximity to each other can significantly impact cancer prognosis because immune cells play a key role in tumor surveillance, suppression, and progression 43. The concept of hot versus cold tumors is crucial in understanding cancer prognosis and response to immunotherapy. This classification is based on the level of immune cell infiltration, particularly cytotoxic CD8+ T cells, within the tumor microenvironment (TME) 44.

Hot tumors are characterized by high levels of immune infiltration, particularly CD8+ T cells, and an inflamed microenvironment with active immune signaling 45. These tumors often express immune checkpoint molecules such as PD-L1, making them more responsive to immune checkpoint inhibitors (ICIs) like anti-PD-1/PD-L1 therapies 46.

Patients with hot tumors generally have better survival outcomes, as their immune systems are actively recognizing and attacking cancer cells 47-51. These tumors often have a high tumor mutational burden (TMB), leading to more neoantigens that immune cells can recognize 52-56.

Because these tumors already have infiltrating T cells, treatments such as checkpoint blockade therapy further enhance the immune response, improving prognosis 57-62.

Cold tumors, lack significant immune cell infiltration, making them immunologically "silent" or non-inflamed. These tumors often have a low TMB, meaning they produce fewer neoantigens, reducing immune recognition. Patients with cold tumors typically have worse survival outcomes, as their tumors evade immune detection and are resistant to immunotherapy 63-65.

Cold tumors often create a physical or biochemical barrier that prevents T cells from entering, such as fibrotic stroma, immunosuppressive cytokines (e.g., TGF-β), or regulatory T cells (Tregs) 66, 67. Since these tumors lack pre-existing immune activation, ICIs are often ineffective without additional interventions.

To turn cold tumors into hot tumors, therapeutic strategies aim to reduce the physical barriers and attract T cells into the tumor microenvironment (TME). Approaches such as vascular normalization, TGF-β inhibition, and stromal remodeling can enhance T-cell infiltration. Additionally, immune checkpoint blockade therapies, such as anti-PD-1/PD-L1 and anti-CTLA-4, help reinvigorate exhausted T cells and improve their spatial distribution. Spatial computational tools can quantify changes in T-cell proximity to tumor cells before and after such treatments, providing insights into the effectiveness of therapeutic interventions. Turning cold tumors into hot tumors by improving T-cell infiltration is directly related to spatial proximity and nearest-neighbor analysis 68.

The fundamental challenge in cold tumors is the lack of functional immune cell infiltration, which results in immune evasion and resistance to immunotherapies 69. Spatial analysis techniques help in understanding and quantifying the distribution of immune cells in tumors, allowing for strategies to improve T-cell proximity to cancer cells and ultimately enhance anti-tumor responses. One of the primary mechanisms behind cold tumors is the formation of an immunosuppressive microenvironment, which includes physical and biochemical barriers preventing T-cell infiltration. Hence, the spatial organization of immune cells is a key determinant of tumor immunogenicity, and nearest-neighbor analysis provides a valuable framework for studying T-cell infiltration dynamics. By leveraging these spatial insights, therapeutic strategies can be designed to reshape the tumor microenvironment, enhancing immune cell proximity and transforming cold tumors into hot tumors for better clinical outcomes 70.

Another key aspect is the interaction between antigen-presenting cells (APCs) and T cells 71-77. Dendritic cells (DCs) play a crucial role in recruiting and activating T cells, and their proximity to T-cell populations within the tumor predicts better immune responses. Nearest-neighbor analysis can reveal whether DCs are optimally positioned to stimulate T-cell responses or whether their spatial arrangement limits immune activation. Enhancing the recruitment of activated DCs to tumors using cancer vaccines or adjuvants can improve T-cell infiltration and conversion of cold tumors into hot tumors.

The spatial proximity of CD8+ T cells to cancer cells further refines this classification. Even within hot tumors, if T cells are excluded from the tumor core and trapped at the tumor margin, their effectiveness is reduced. Nearest-neighbor analysis helps quantify these interactions, showing that a shorter distance between CD8+ T, CD4+ Tcells and tumor cells correlates with better survival outcomes 18.

This study by Azimi et al. 18, identifies the median distance between CD8+ T cells, CD4+ T cells, and tumor cells as a crucial spatial biomarker associated with patient survival after treatment. Lower distances between these immune cells and tumor cells correlate with better prognosis, as demonstrated through Kaplan-Meier survival analyses using disease-free survival (DFS) data. The data differentiates cancer cores into "Isolated" (greater distances) and "Non-Isolated" (smaller distances) groups. Cancer cores in the Non-Isolated group exhibit higher expression of Caspase-3, cleaved Caspase-3, Caspase-8, Ki67, and HLA-1, suggesting these biological markers are linked to closer immune-tumor interactions. It also illustrates a ClusterMap for the discovery cohort, highlighting expression patterns of key tumor biomarkers (Caspase-3, Ki67, C-myc, GLUT-1, HLA-1) and their spatial relationships with cytotoxic and regulatory T cells. This visualization underscores the significance of spatial immune-tumor interactions. Data shows that patients grouped into two distinct risk clusters (Cluster 1: high risk; Cluster 2: low risk) based on these spatial and biological markers demonstrate substantial differences in survival outcomes, further supporting the prognostic value of these spatial features.

By mapping the nearest-neighbor relationships between immune cells and tumor cells, it is possible to identify spatial patterns associated with treatment response and resistance. For example, in successful immunotherapy responses, CD8+ T cells move closer to tumor cells, reducing the average nearest-neighbor distance, whereas in resistant tumors, immune cells remain spatially distant. These insights can guide the development of combination therapies that optimize T-cell positioning within tumors.

The hot vs. cold tumor paradigm is a powerful framework for predicting cancer prognosis and response to immunotherapy. While hot tumors generally indicate a better prognosis, cold tumors require targeted interventions to enhance immune infiltration and improve patient outcomes. The spatial distribution of immune cells is a crucial factor in determining whether a tumor can effectively be turned from cold to hot, shaping treatment strategies in precision oncology.

## Nearest Neighborhood Analysis, Cell-Cell Interaction Map and Prognosis

Nearest Neighbor Analysis (NNA) and Cell-Cell Interaction Maps (CCIM) offer deeper insights into immune-tumor dynamics by quantifying the spatial relationships between tumor cells, immune cells, and stromal components. These methods enable researchers to measure key parameters such as immune cell clustering, tumor cell proximity to T cells, and the presence of immune-privileged niches within tumors. By integrating these spatial metrics with clinical outcomes, researchers can derive prognostic signatures that predict disease progression and therapeutic response more accurately than conventional molecular biomarkers.

The interactions between epithelial and stromal cells are fundamental to tissue homeostasis and cancer progression. Epithelial cells, which form continuous sheets lining surfaces, rely on direct cell-cell adhesions to maintain barrier integrity and polarity. In contrast, stromal cells—including fibroblasts, pericytes, and extracellular matrix-producing cells—engage in dynamic, often paracrine-mediated interactions that provide structural scaffolding and modulate epithelial behavior. These differences in interaction modes yield distinct consequences: epithelial cell contacts predominantly regulate tissue architecture and proliferation control, while stromal cell interactions shape the biochemical and mechanical landscape of the tumor microenvironment, influencing invasion and metastasis 78.

The spatial interactions of epithelial and hematogenous cell populations exhibit distinct biological and functional features. Epithelial cells maintain stable junctional complexes that regulate tissue integrity and polarity, while hematogenous (blood-derived) cells, such as circulating immune cells, engage in transient interactions for immune surveillance and infiltration. These differences manifest in diverse consequences: epithelial cell interactions primarily influence structural homeostasis and local signaling networks, whereas hematogenous cells dynamically shape the tumor microenvironment through trafficking and immune modulation. Recognizing these distinctions is crucial to understanding how tumor progression exploits these divergent interaction paradigms 79.

Interactions between tumor cells and immunoreactive cells, such as T cells or natural killer cells, embody critical crosstalk in the tumor microenvironment. While tumor cells typically leverage direct cell-cell interactions to promote immune evasion, immunoreactive cells utilize these interactions to mount cytotoxic or regulatory responses. The balance of these interactions determines immune suppression or activation within the tumor niche. Understanding these nuanced interactions underscores the potential of targeting cell-cell contacts for immunotherapeutic interventions 80.

Beyond binary cell interactions, the tumor microenvironment is defined by a rich interplay among epithelial, stromal, hematogenous, and immune cells. Each cell type contributes unique molecular cues and mechanical properties that collectively shape tumor behavior. For instance, stromal fibroblasts provide structural support and paracrine signals, while immune and vascular cells regulate infiltration and angiogenesis. By differentiating these multifaceted interactions, we gain a clearer picture of the ecosystem-level dynamics of cancer progression^81^.

Using surgical specimens, a retrospective examination of 22 pleural mesothelioma patients treated with nivolumab in various institutions was carried out in study by Yin et al. 82. The response group's density of CD8+ T cells and total T cells was noticeably greater than that of the nonresponse group. While regulatory T cells were found farther away from tumor cells in the response group than in the nonresponse group, CD8+ T cells were more grouped and situated nearer to tumor cells. While regulatory T cells' proximity to tumor cells was linked to a worse response to nivolumab, CD8+ T cells' high density and spatial proximity to tumor cells were linked to a better response. This suggests that the unique TME landscape may be a potential predictor of immune checkpoint inhibitors (ICIs) efficacy in pleural mesothelioma.

Sorin et all. 83 spatially analyzed the tumor immune microenvironment (TIME) of lung adenocarcinoma (LUAD) using imaging mass cytometry (IMC), finding characteristics that were significantly predictive of recurrence in early-stage disease6. Notably, we found that enhanced survival in LUAD patients was linked to certain interactions between B cells and CD4+ T cells. However, when regulatory T cells were discovered near B cells and CD4+ T cells, this survival benefit was eliminated. Furthermore, they found that patient-specific variables like age, sex, or smoking status had an impact on a number of TIME parameters, especially within the myeloid compartment.

In order to quantify 14 TIIC subgroups in situ, the authors 84 used multiplex immunohistochemistry staining technique on 190 colorectal (CRC) samples. The computational process phenoptr was used to calculate the separation between immune and cancer cells. Myeloid lineage cells were found closest to tumor cells, and the epithelial compartment was enriched in MPO+ neutrophils and CD68+IDO1+ tumor-associated macrophages (TAMs). All other cells, with the exception of CD68+CD163+ TAMs, were positively correlated with a good prognosis. The distance between TIICs and tumor cells has a significant impact on their prognostic predictive capacity. Correlation analysis demonstrated the cooperation of B cells, CD68+IDO1+TAMs, and T lineage cells in generating an efficient immune response, whereas unsupervised clustering analysis separated colorectal cancer into three categories with different prognostic outcomes.

In a study by Ebia et al. 85, two groups of 22 stage I/II PDAC patients were created: Standard responders (n = 11) whose overall survival (OS) was greater than two years, and poor responders (n = 11) whose OS was less than two years. To determine cell phenotypes, multiplex IHC was used on tissues utilizing several biomarkers (CD8, CXCR4, CD66b, FAK, FAP, CD68, CSF1R, and EPCAM). According to the study, the spatial distance between tumor microenvironment immune cells in pancreatic ductal adenocarcinoma (PDAC) may have an impact on clinical outcomes. CD8+ T cells that were further from M2 and M1 macrophages and closer to FAK+ tumor cells, CXCR4+ TANs, and CAFs had longer survival times. The biological and prognostic relevance of cell distance among TME phenotypes may be better understood by larger cohort investigations, which might potentially affect how well immunotherapy works.

The effectiveness of neoadjuvant treatment (NAT) for breast cancer (BC) is significantly influenced by the spatial proximity of cytotoxic T lymphocytes to tumor cells according to the study by Liang et al. 86. In this work, the authors assessed whether treatment results for different BC subtypes may be predicted by the presence of CD8+ T cells and other immune cells close to cancer cells. Using multiplex immunofluorescence (mIF) and immunohistochemistry (IHC), they examined pre- and post-NAT biopsies from 104 BC patients to evaluate the distribution of immunological markers, including as CD8+ T cells, CD68+ macrophages, and FoxP3+ regulatory T cells. Regardless of tumor subtype or NAT regimen, our results showed that a larger percentage of CD8+ T cells within 20 µm of cancer cells (N20-CD8+ T cells) was highly associated with enhanced pathological complete response (pCR), disease-free survival (DFS), and overall survival (OS).

In a different study 87, researchers created a multiplex immunohistochemistry (mIHC) antibody panel to quantitatively examine leukocyte lineages, with a particular emphasis on NK cells and their characteristics, in two separate cohorts of patients with breast cancer (n = 26 and n = 30). Spatial analysis showed different NK cell characteristics in relation to their proximity to neoplastic tumor cells that were connected with HER2 status, although NK cell density and phenotype did not seem to be affected by HER2 status. Multiple distinct neighborhood compositions surrounding NK cells were identified by spatial cellular neighborhood analysis. NK cells from HER2-tumors were more commonly found proximal to neoplastic tumor cells, while NK cells from HER2+ tumors were more frequently found proximal to CD3+ T cells.

Multiplex immunofluorescence was used to stain single sections of diagnostic biopsies from 72 oropharyngeal squamous cell carcinoma (OPSCC) patients (CD8, PD1, PD-L1, CD68) in study by Tsakiroglou et al 88. After automated regions-of-interest selection and multispectral scanning, the Hypothesised Interaction Distribution (HID) approach measured the spatial proximity of cells. The predictive importance of co-localized cells (within 30 μm) in patients stratified by HPV status was examined in order to assess the method's applicability. In patients with HPV negative OPSCC (n = 31), high frequencies of proximal CD8+ and PD-L1+ (HR 2.95, p = 0.025) and PD1+ and PD-L1+ (HR 2.64, p = 0.042) cells were predictive of poor overall survival.

According to the authors 89, TILs have been linked to a lower chance of recurrence in cases with HPV(+)OPSCC, or human papillomavirus-associated oropharyngeal squamous cell carcinoma. Imaging mass cytometry was used to examine primary and lymph node (LN) tumor tissues that were paraffin-embedded and formalin-fixed from ten progressors (cases) and ten matched non-progressors (controls). Machine learning was used to quantify immune, stromal, and tumor cells from specific areas of interest (ROIs). Analysis of niches, cell-cell interactions, and nearest neighbors were done. The proportion of T cells, CD8+ T cells, innate cells, immune cells, and lymphocytes was considerably higher in controls in primary ROIs. In primary tissues under control, the average distances between T cells and the closest B cells as well as between lymphocytes and the closest tumor cells were reduced.

Ten recurrent cellular neighborhoods—a group of local TME features with distinct cell components—were found through spatial analysis in a study by Mi et al.^90^. Positive clinical outcomes were substantially linked with the relative spatial colocalization of SMAhi fibroblasts and tumor cells as opposed to B cells. The authors predicted the response of a different cohort of patients in the NeoTRIP clinical trial to treatment based on baseline TME features with high accuracy (mean area under the receiver operating characteristic curve of 5-fold cross-validation = 0.71) by using a deep learning model trained on engineered spatial data. In addition to suggesting new imaging-based biomarkers for the development of treatments in the setting of TNBC, these findings further support the idea that the TME architecture is organized in cellular compositions, spatial structures, vascular biology, and molecular profiles.

The authors in the study 91 profiled spatial interactions in non-small cell lung cancer (NSCLC) patients who later underwent PD1 axis treatment using an analytical pipeline for highly multiplexed CODEX data. In line with their placement inside stromal and peripheral tumor-margins, they discovered that regulatory T cells (Tregs) are abundant in non-responding patients. While macrophages were more commonly identified in close proximity to HLADR+ tumor cells (p = 0.01) in responding patients, proximity-based interactions between Tregs and both monocytes (p = 0.009) and CD8+ T cells (p = 0.009) were more common in non-responding patients. Analysis of cellular neighborhoods revealed that CD8+ T cells (p = 0.03) in HLADR+ tumor neighborhoods and macrophages (p = 0.003) and effector CD4+ T cells (p = 0.01) in mixed tumor neighborhoods were linked to positive clinical outcomes.

The authors in 92 investigate the effects of endoplasmic reticulum (ER) stress on tumor-related cells, including immune cells, endothelial cells, and cancer associated fibroblasts (CAFs), on patient outcomes in clinical specimens. The study showed that CAFs and immune cells mostly experience ER stress when they are close to tumor cells in PDAC patient tissue. Poor patient survival was associated with immune cells expressing high levels of CHOP. When CAFs and immune cells were near tumor cells (< 20 μm), they were more likely to express BiP or CHOP. Better patient survival was associated with higher levels of CHOP expression in CAFs around tumor cells.

The authors in the study 93 used multiplex immunohistochemistry to examine the main tumors and lymph nodes of 271 non-small cell lung cancer (NSCLC) patients. The results indicated that there were four activation states for CD8+TRM. The densities of PD-1-TIM-3+CD8+TRM3 and PD-1+TIM-3+CD8+TRM4 were significantly greater in the invasive margins of locally progressed non-small cell lung cancer. In more advanced lesions, there were fewer interactions between CD8+TRM and tumor cells. In patients with early lung adenocarcinoma and squamous carcinoma, respectively, recurrence was independently linked to decreased PD-1+TIM-3-CD8+TRM2 contacts with tumor cells and enhanced PD-1+TIM-3+CD8+TRM4 interactions with tumor cells. These findings highlight the significance of CD8+TRM spatial dynamics in prognosis by indicating a relationship between recurrence risk and CD8+TRM activation state and distribution in early-stage NSCLC.

## Conclusions

Cancer is a complex and heterogeneous disease, influenced not only by genetic mutations but also by the spatial organization of cells within the tumor microenvironment (TME). The arrangement and interactions between tumor cells, stromal cells, and immune infiltrates can significantly impact disease progression, response to therapy, and overall patient prognosis. As a result, understanding the spatial architecture of tumors has become a crucial area of research in oncology. Traditional histopathology has long recognized the prognostic value of tumor architecture, but recent advancements in computational pathology and spatial biology have provided more precise and quantitative methods to study these spatial relationships.

Several key metrics are used in spatial analysis for cancer prognosis:

• Nearest Neighbor Distance (NND): One such approach is nearest neighbor analysis (NNA), a statistical method used to quantify the spatial distribution of cells in tissue samples. NNA measures the distances between a given cell and its closest neighboring cells, allowing researchers to assess patterns of clustering, dispersion, or randomness. This method is particularly useful in oncology, where the spatial arrangement of immune cells relative to tumor cells can provide key insights into immune evasion mechanisms, treatment resistance, and patient survival outcomes. This metric is useful for evaluating immune cell infiltration, tumor cell clustering, and spatial dispersion of different cell types.

• Ripley's K-Function: A statistical measure used to analyze clustering patterns at different spatial scales. It helps determine whether cells are randomly distributed, clustered, or dispersed within a tumor sample.

• Voronoi Tessellation: A computational approach that partitions space into regions based on proximity to a set of points. In cancer research, Voronoi diagrams help identify tumor cell niches and assess the spatial dominance of specific cell populations.

• Graph-Based Methods: These methods construct spatial graphs where nodes represent cells and edges reflect their spatial relationships. Graph theory is particularly useful for modeling complex tumor architectures and analyzing immune-tumor interactions.

Proximity analysis, a broader category of spatial statistics, extends beyond individual neighbor distances to examine the overall spatial organization and interaction networks within the tumor microenvironment. Proximity analysis has been widely used to explore tumor-immune interactions and their prognostic implications. For instance, studies have shown that the spatial positioning of cytotoxic CD8+ T cells relative to tumor cells is a strong predictor of survival in multiple cancer types, including colorectal, breast, and lung cancer. Tumors with a high density of T cells in close proximity to cancer cells often exhibit better immune surveillance and improved patient outcomes.

By leveraging these spatial insights, therapeutic strategies can be designed to reshape the tumor microenvironment, enhancing immune cell proximity and transforming cold tumors into hot tumors for better clinical outcomes.

These analyses enable researchers to define tumor-immune spatial niches and predict their functional implications.

## Figures and Tables

**Table 1:** Computational methods for measuring cell spatial connections in order to examine tumor-immune interactions

Method	Description	Typical Application
Nearest Neighbor Distance	Measures closest cell distances	Immune infiltration, tumor heterogeneity
Voronoi Tessellation	Divides tissue into polygons	Cellular niche analysis
Ripley's K-function	Measures clustering/dispersion	Clonal expansion, immune exclusion
Graph-based Models	Networks of cell-cell interactions	Immune-tumor interplay, spatial niches
